# Early Detection of *Mycobacterium avium* subsp. *paratuberculosis* Infected Cattle: Use of Experimental Johnins and Innovative Interferon-Gamma Test Interpretative Criteria

**DOI:** 10.3389/fvets.2021.638890

**Published:** 2021-05-14

**Authors:** Sara Corneli, Antonella Di Paolo, Nicoletta Vitale, Martina Torricelli, Linda Petrucci, Carla Sebastiani, Marcella Ciullo, Ludovica Curcio, Massimo Biagetti, Paola Papa, Silva Costarelli, Monica Cagiola, Alessandro Dondo, Piera Mazzone

**Affiliations:** ^1^Istituto Zooprofilattico Sperimentale dell'Umbria e delle Marche “Togo Rosati”, Perugia, Italy; ^2^Istituto Zooprofilattico Sperimentale del Piemonte Liguria e Valle d'Aosta, Torino, Italy

**Keywords:** *Mycobacterium avium* subsp. *paratuberculosis*, Johne's disease, bovine paratuberculosis, IFN-γ test, purified protein derivatives, Johnin PPD, interpretative criteria, cattle

## Abstract

Paratuberculosis (PTB), also known as Johne's disease, is a chronic proliferative enteritis of ruminants caused by *Mycobacterium avium* subsp.*paratuberculosis* (MAP). To date, PTB diagnosis, based on serology, fecal culture, and real-time polymerase chain reaction, has identified animals in advanced stages of infection. To detect MAP infection in animals earlier, the interferon-gamma (IFN-γ) test may be applied. This assay detects cytokines produced by T-lymphocytes of infected subjects after stimulation with purified protein derivatives (PPDs), extracted from *Mycobacterium bovis* (MB) and from *M. avium* (MA). The study involved three bovine herds: one PTB-infected herd, one PTB-free herd, and one with an outbreak of bovine tuberculosis. The IFN-γ test was performed on 235 animals, using bovine PPD (PPDB), avian PPD (PPDA), and three experimental PPD Johnins (PPDJs) extracted from a synthetic liquid medium culture of MAP (PPDJ A, B, and C), to assess early MAP detection and avoid false reactions to MB. Furthermore, IFN-γ results were evaluated using 12 interpretative criteria (ICs), based on the differences and ratio between PPD optical density (OD) and IFN-γ basal OD values after lymphocytic stimulation. IC accuracy was expressed as area under the receiver operating characteristic curve. Through a longitudinal study, PPDJs proved to be specific and sensitive in the detection of MAP-infected animals. Among the evaluated ICs, six showed the best performance in terms of accuracy (*p* < 0.0001), highlighting PTB subclinical infections. In particular, the two best criteria reached sensitivity values of 100% [confidence interval (CI) 95%, 94.1–100%] with a specificity of 91.8% (CI 95%, 81.9–97.3%) and sensitivity levels of 80.6% (CI 95%, 69.1–89.2%) with a specificity of 100% (CI 95%, 94.1–100%). Thus, the IFN-γ assay proved to be a useful diagnostic tool to identify early subclinical MAP-infected animals, in order to manage infected cattle or those exposed to MAP and to monitor younger calves within a herd. Furthermore, the IFN-γ test can be considered an additional test to avoid the introduction of MAP-infected animals, especially in herds where disease has already been eradicated and preservation of the health status is required to maintain the PTB certification level.

## Introduction

*Mycobacterium avium* subsp. *paratuberculosis* (MAP) is a slow-growing mycobacterium (1) and the causal agent of Johne's disease (JD) or paratuberculosis (PTB), a chronic inflammatory bowel disease seen in farmed ruminants and wildlife species worldwide (2, 3). Infected animals may shed MAP through their feces, and the live bacteria can survive in pastures (4) for a long time, representing a risk to other animals and even humans (5, 6). In fact, MAP may also act as a zoonotic agent in some human diseases (7–10). In particular, for more than a century, it has been associated with Crohn's disease, a chronic inflammatory bowel disease characterized by transmural inflammation and granuloma formation. Recently, other diseases have been associated with MAP, such as sarcoidosis, Blau syndrome, type 1 diabetes, Hashimoto's thyroiditis, and multiple sclerosis (9, 11–16). Regarding the transmission sources, MAP may contaminate food for human consumption, such as dairy and meat products, infant formula (17–19), and water (6, 20); and MAP seems to resist pasteurization treatment of milk at 72°C for 15 s [high-temperature, short-time (HTST) pasteurization] (21–23).

JD causes major economic losses to the global dairy industry due to reduced milk production (24), weight loss, infertility, pre-mature culling, and increased cow replacement costs (25, 26). The prevalence of MAP-infected farms in countries with advanced animal husbandry is growing rapidly and varies worldwide between 7 and 55% (27–29). Control programs to manage PTB in cattle and sheep herds have been adopted and in some cases re-adopted over the past couple of decades, in different countries, such as Australia, the USA, the Netherlands, Japan, and Denmark (28–32). The effectiveness of several recent control programs is yet to be demonstrated, since no countries have yet been able to eradicate the infection and there is no international agreement on PTB eradication plans (29, 30, 33).

A major problem in PTB control is the identification of animals in the early stages of MAP infection. The animals become most frequently infected at a young age, rarely *in utero*, but more often as newborns (34–36). The fecal–oral route is the main route of transmission, including the ingestion of contaminated feed, fodder, milk, and colostrum (35, 37, 38).

The initial host defense against MAP infection is mediated by the lymphocyte T-helper 1 (Th1) response, characterized by the production of IFN-γ and other pro-inflammatory cytokines (39–41). Nevertheless, MAP can use its evasion mechanisms and can survive by interfering with the host immune response (42). In particular, MAP is captured and processed by macrophages, in which mycobacteria can replicate and avoid phagolysosome maturation (43, 44). This step is followed by the activation of the cell-mediated immune response, which attempts to contain MAP infection (45). Humoral immune response, mediated by lymphocyte Th2, also appears in mycobacterial infection but generally only appears late when the disease is already established (46). This is an oversimplification of what happens in animals during MAP infection, since it has been demonstrated that an overlap between Th1 and Th2 responses could exist (47, 48). In the animals that “lose the battle against infection,” two or more years can pass before the appearance of the first clinical symptoms, and the evolution of the infection is not obvious in all subjects (49). Clinical disease is characterized by intermittent diarrhea, progressive weight loss, inappetence, and death (36, 45). The progression of the infection depends on both the containment action of innate and cell-mediated immunity (CMI) and is related to host genetics (50) and environmental factors. Therefore, PTB can be defined as a conditioned disease (43, 49, 51). The diagnosis of PTB, based on enzyme-linked immunosorbent assay (ELISA) for antibody detection, MAP fecal culture, and real-time quantitative polymerase chain reaction (qPCR) to detect MAP DNA, is particularly difficult (52). In fact, during the initial and subclinical stages, in MAP-infected animals, specific antibodies are absent or present at low levels, while MAP can be excreted intermittently at low concentrations (49, 53–55). When the infection advances, circulating antibodies gradually increase and are easily detectable by ELISA; however, they have no protective effect (46, 56, 57). Generally, animals that are positive for PTB ELISA are in advanced stages of infection; therefore, diagnostic tests based on measuring CMI, such as IFN gamma-release assays (IGRAs), could be more suitable for revealing subclinical stages of infection (48, 49, 57–62). IGRAs, which are used for the diagnosis of mycobacterial infections, consist of the quantification of IFN-γ released after mycobacterial antigen stimulation of peripheral blood lymphocytes. The test is performed using commercial ELISA kits to detect the amount of cytokines produced and secreted by T-cells of infected animals in culture supernatants (63, 64). The IFN-γ assay, developed in Australia in the late 1980s, is an *in vitro* blood test used as an ancillary test in combination with the skin test for the diagnosis of bovine tuberculosis (bTB) due to *Mycobacterium bovis* (MB) (63–67). Recently, it has also been used for the early diagnosis of PTB (60). As reported in the literature, the specificity (Sp) of the method varies from 67 to 94%, while the sensitivity (Se) varies from 13 to 85%, depending on the type and quantity of purified protein derivatives (PPDs) and, particularly, on the interpretative criteria (ICs) adopted for the tests (57, 58, 60). The antigens normally used during the stimulation phase of lymphocytes in the IFN-γ test are the traditional bovine and avian tuberculin PPDs extracted from MB AN5 (PPDB) and *M. avium* D4ER (PPDA), respectively. Johnin (PPDJ), similar to the other tuberculins, is a crude PPD obtained from a MAP culture in a synthetic liquid medium, inactivated by heat treatment, precipitated with trichloroacetic acid, and re-suspended in phenol and glycerin (57, 68).

In the present study, we used three different experimental PPDJs, which are produced in Italy at Istituto Zooprofilattico Sperimentale dell'Umbria e delle Marche “Togo Rosati” (IZSUM) and described by Corneli et al. (69).

The commercial diagnostic ELISA tests available today that are used for bTB have not been validated by the manufacturer for the diagnosis of PTB (57, 58). The interpretation of the values of secreted IFN-γ, expressed as absorbance values in optical density (OD) and relative cutoff points, is crucial for defining the outcome of the IFN-γ test and thus to clarify the state of mycobacterial infection in the animal (58, 70–74). However, there is a substantial difference between the use of the IFN-γ test for bTB diagnosis and the use of the IFN-γ test for PTB diagnosis. In the first case, the aim of the test is to detect the infected animal earlier than the skin test can (63) to facilitate prompt slaughter of the positive animals, according to the “test and cull” strategy. By contrast, in the case of PTB, the IFN-γ test still aims to identify the infected animal, but a different destination can be assigned to this animal depending on the prevalence of the disease in the herd. In a herd where the prevalence is low or PTB is not present, with an advanced health certification for PTB, the animal has to be removed from the herd. Conversely, in a herd with a high prevalence of PTB, animals with a positive reaction to the IFN-γ test for MAP infection diagnosis might be at risk of MAP shedding, and therefore, animals need to be checked more often than others. As reported by several authors (49, 75), these cattle could also include the animals able to contain the evolution of the MAP infection, without ever developing the subclinical and clinical forms of PTB, and therefore, they represent a genetic resource to be preserved and enhanced.

The aim of this longitudinal study was to evaluate the performance of three new experimental PPDJs and their potential application, in association with Italian PPDA and PPDB, in the IFN-γ test for the early detection of animals infected with MAP.

## Materials and Methods

### Production of Purified Protein Derivative Johnin

For the production of the new Italian Johnin, 20 Italian MAP strains were genotyped by amplification of mini- and microsatellite loci at the Italian National Reference Center for PTB (76). Two field strains, identified as strain A (used for PPDJA) and strain B (used for PPDJB), were selected based on their geographical distribution, growth characteristics, and protein yield. The MAP American Type Culture Collection (ATCC) strain 19698 represented strain C and was used for a third batch of Johnin (PPDJC) only as a production control and for methodical optimization. The three MAP strains were cultured for 4 months at 37°C in Watson–Reid modified broth, and then the bottles were autoclaved at 100°C for 3 h. The cells were removed, and the proteins were extracted by precipitation with trichloroacetic acid. The precipitate was then washed and dissolved in phosphate phenolate buffer and glycerine, with a final protein concentration of 1 mg/ml, as required for PPDB by the European regulation Annex B, Directive 64/432/EEC (77), and European regulation (EC) No. 1226/2002 (78).

### Field Trial

#### Animal Population Characteristics and Ethics Statement

All samples were assessed as per the periodic tests required by the Italian National Health Programs (78–80) and during farmers' voluntary health controls for PTB, provided by the Italian National Guidelines (81).

A total of 235 cattle from farms in central Italy were enrolled in the study and divided into three groups, as follows:

- The first group consisted of 87 dairy cattle from three bTB Officially Free (OF) herds, where clinical cases of PTB had been reported.
- The second group consisted of 61 beef cattle from a bTB OF herd without PTB cases, and the herd had tested negative for serological tests in the last 4 years.
- The third group included 87 beef cattle from a bTB-positive herd with an ongoing outbreak when the study was performed.

#### Paratuberculosis Status Assessment

Each animal from the bTB OF herds with previous PTB cases or without PTB was assessed in parallel to traditional PTB tests, as follows:

- PTB ELISA test on serum (“ID Screen^®^ paratuberculosis Indirect”—IDVet Innovative Diagnostics, Montpellier, France) in accordance with the manufacturer's instructions;
- MAP isolation on selective solid media following the OIE terrestrial manual (82);
- IS900 qPCR for MAP DNA detection from feces (83, 84) in fast mode (85).

As part of the longitudinal study, cattle were monitored for 4 years to check the evolution of their health status in relation to PTB, and animals were considered positive for PTB if at least one of the three tests (ELISA and/or qPCR and/or MAP culture) yielded a positive result.

### Interferon-γ Assay and Interpretative Criteria

#### Whole Blood *in vitro* Stimulation and Interferon-γ Detection

Jugular blood samples were collected and delivered to the laboratory within 12 h at room temperature. The heparinized blood samples of each animal were dispensed in aliquots of 1 ml and stimulated, respectively, with phosphate-buffered saline (PBS 0.01 M, pH 7.2), used as a nil control antigen, which represented the IFN-γ basal value in the single animal (PBS); 10 μg of Italian PPDB and 10 μg of Italian PPDA; 20 and 10 μg of the three experimental PPDJs (strains A, B, and C, respectively), with two different dilutions 1:5 and 1:10; Pokeweed Mitogen (PWM; Thermo Fisher Scientific™, Waltham, MA, USA) included at a final concentration of 1 μg/ml, as a positive control of lymphocyte viability. IFN-γ secretion was evaluated using the Bovigam IFN-γ kit (Thermo Fisher Scientific™) in the plasma collected after 24 h of incubation at 37 ± 1°C in 5% CO_2_. The obtained values were expressed in units of OD measured at 450 nm (OD 450 nm).

#### Interferon-γ Interpretative Criteria

For the PTB IFN-γ performance evaluation, a comparison among PPDB, PPDA, and PPDJ was carried out, applying differences or ratios among the PPD OD values obtained. For each criterion, different cutoffs were applied to interpret the results. In particular, 12 possible ICs were adopted in the first and second groups to assess the presence or absence of MAP (MAP reactive or MAP negative) and to evaluate the stage of MAP infection. In the third group, to verify the test performance and reliability in the presence of another mycobacterial infection, three of the best criteria, chosen among those evaluated, were adopted. The ICs, shown in Table 1, were applied to all PPDJs (A, B, and C) at two dilutions (1:5 and 1:10) in association with Italian bovine and avian PPDs. Briefly, from the first to fifth criteria, we considered the difference between the PPDs OD values obtained after lymphocyte stimulation and the OD basal value with three different cutoff values (if PPDA or PPDJ – PBS > 0.05; 0.1; 0.2 = MAP infection) and twice the OD basal value at two different cutoff values (if PPDA or PPDJ – 2 ^*^ PBS > 0; 0.04 = MAP infection). From the sixth to ninth criteria, we considered the ratio between the PPD OD values obtained (PPDB or PPDA/PPDJ ≤ 0.09 = MAP infection; PPDJ/PPDB or PPDA > 1 = MAP infection). For the 10th criterion, we compared the ratio between PPDs (PPDB/PPDA > PPDB/PPDJ = MAP infection); and finally, for the 11th and 12th criteria, we considered the difference between PPDA and PPDJs and two different cutoff values (PPDJ – PPDA > 0.05; 0.1 = MAP infection).

**Table 1:** Interpretative criteria of the IFN-γ test and cutoff values applied in the study for the diagnosis of *Mycobacterium avium* subsp. *paratuberculosis* (MAP) infected cattle.

	**Interpretative criteria**	
**1**	Difference between PPDAv and PBS value > 0.05	If PPDAv – PBS > 0.05 = MAP
	Difference between PPDJa or Jb and PBS value > 0.05	If PPDJ – PBS > 0.05 = MAP
**2**	Difference between PPDAv and PBS value > 0.1	If PPDAv – PBS > 0.1 = MAP
	Difference between PPDJa or Jb and PBS value > 0.1	If PPDJ – PBS > 0.1 = MAP
**3**	If the difference between the PPDAv value and PPDJa or Jb and PBS value is >0.2, the animal is considered MAP reactive	PPDAv – PBS > 0.2 = MAP
		PPDJ – PBS > 0.2 = MAP
**4**	If the reaction to Italian PPDAv or PPD Ja or Jb is two-fold the PBS value, the animal is considered MAP reactive	PPDAv – (2 ^*^ PBS) > 0 = MAP
		PPDJ – (2 ^*^ PBS) > 0 = MAP
**5**	Difference between PPDAv and twice the PBS value > 0.04	If PPDAv – (2 ^*^ PBS) > 0.04 = MAP
	Difference between PPDJa or Jb and twice the PBS value > 0.04	If PPDJ – (2 ^*^ PBS) > 0.04 = MAP
**6**	First level:	If PPDBov and PPDJ > 2 ^*^ PBS then apply PPDBov/PPDJ
	Second level:	If PPDBov/PPDJ ≤ 0.9 = MAP and if PPDBov/PPDJ ≥ 1.1 = MB
		If PPDBov/PPDAv ≤ 0.9 = MAP and if PPDBov/PPDAv ≥ 1.1 = MB
	In case of intermediate values, the result is inconclusive or not discriminant (ND).	
**7**	First level:	If PPDAv and PPDJ > 2 ^*^ PBS then apply PPDBov/PPDJ
	Second level:	If PPDAv/PPDJ ≤ 0.9 = MAP and If PPDAv/PPDJ ≥ 1.1 = MA
	In case of intermediate values, the result is inconclusive or non-discriminant (ND).	
**8**	PPDJa or Jb and PPDBov ratio	If PPDJ/PPDBov > 1 = MAP
**9**	PPDJa or Jb and PPDAv ratio	If PPDJ/PPDAv > 1 = MAP
**10**	Comparison of the ratios between PPDBov and PPDAv and between PPDBov and PPDJa or Jb ratio	If PPDBov/PPDAv > PPDBov/PPDJ = MAP
**11**	Difference between PPDJa or Jb and PPDAv > 0.1	If PPDJ – PPDAv > 0.1 = MAP
**12**	Difference between PPDJa or Jb and PPDAv > 0.05	If PPDJ – PPDAv > 0.05 = MAP

PPD, purified protein derivative; PPDBov, bovine PPD; PPDAv, avian PPD; PPDJ, Johnin PPD; PPDJa, Johnin PPD from strain A; PPDJb, Johnin PPD from strain B; PBS, phosphate-buffered saline. The asterisk symbol (^*^) is used to indicate the mathematical operation of multiplication.

For every criterion, a maximum threshold of the basal value (PBS ≤ 0.150 OD) was introduced to verify the eligibility of the sample. As reported in the literature (73, 86), this additional quality control is already used in the diagnosis of bTB to exclude animals with high basal values due to pre-existing pathologies or to avoid contaminated blood samples. This quality control (PBS ≤ 0.150 OD) was validated by the Istituto Zooprofilattico Sperimentale del Piemonte, Liguria e Valle d'Aosta Laboratory, with the sixth criterion applied to eradicate bTB in Piedmont from 2004 to 2016, to obtain European official tuberculosis-free status (87), and it is still being used (88–90).

The sixth criterion is currently also adopted in the IZSUM for the official diagnosis of bTB.

### Statistical Analysis

The performance of the PPDJs in the 12 IFN-γ test ICs was evaluated on the OD values obtained from a total of 235 cattle, including 87 cattle from PTB affected herds, 61 cattle from a bTB OF herd, without PTB cases in the last 4 years, and another group of 87 cattle from a bTB-positive herd.

To establish the diagnostic accuracy and to compare the diagnostic efficiency of PPDJs, a receiver operating characteristic (ROC) curve analysis was performed. For each criterion, the Se, Sp, accuracy, area under the ROC curve (AUC), and Youden index were calculated. Differences in accuracy, Se, and Sp among the criteria were assessed using a binomial exact test.

The association statistics, the AUC with its standard error, and a confidence interval (CI) for each model were calculated. Differences between the AUC for PPDJA, PPDJB, and PPDA were performed by ROCCONTRAST statements using the non-parametric approach of DeLong (91).

For the purpose of the study, Se was defined as the proportion of samples with positive results from the expected true-positive animals, while Sp was defined as the proportion of samples with negative results to the expected true-negative animals.

The Se of PPDJ (A, B, and C) in the detection of MAP-infected subjects was calculated considering positive animals with a positive outcome in ELISA and/or qPCR and/or fecal culture. The Sp of PPDJ (A, B, and C) was calculated considering negative animals with a negative outcome in ELISA, qPCR, and fecal culture from the herd PTB-free for at least 4 years.

The PPDJ OD distribution was analyzed using histograms, and comparisons between dilutions and strains are shown in box plot graphs.

Two types of analysis of variance were performed using Proc generalized linear models and SAS software v. 9.2 to evaluate the “dilution factor” (1:5 and 1:10) and “strain factor” (A, B, and C) of the PPDJs, in particular if the PPDJ OD concentration values were different among the three strains and between the two dilutions.

## Results

### Paratuberculosis Status Assessment

Animals belonging to the three groups underwent traditional PTB tests: ELISA PTB, qPCR, and MAP isolation from feces (57). Each subject was considered positive for PTB if at least one of the three tests was positive.

In the first group, among the 87 cattle coming from bTB OF herds with previous PTB cases, 71 were positive for at least one of the traditional tests and 16 were negative to all the PTB tests. In particular, considering only the serological test, 68 subjects were positive, three were doubtful, and 16 were negative. Eighteen cattle were positive for real-time PCR from feces, and 28 were positive for MAP fecal culture. In the next 4 years, among the 16 subjects negative for the three PTB traditional tests at the first examination, five became positive during the follow-up (Supplementary Table 1). Specifically, one subject became positive for the culture test 3 months later, one was positive for ELISA and MAP isolation 10 months later, and two cattle became positive for ELISA (Supplementary Figure 1).

In the second group, among 61 cattle belonging to an OF bTB herd and without cases of PTB in the last 4 years, 100% of the animals were negative using conventional diagnostic tests.

The third group consisted of 87 cattle from a herd in which bTB positivity to the skin test was registered and then confirmed by the isolation of MB. In the first sampling of the study, the bTB outbreak was still present and PPDA reactivity was found in the comparative skin test. For this reason, the IFN-γ test and ELISA PTB test were performed in support of the official bTB diagnosis to avoid false-positive bTB outcomes in eventually PTB-positive animals. The herd was monitored for another 2 years until the bTB-free status was regained and all animals were negative in the two ELISA tests performed annually.

### Evaluation of Purified Protein Derivatives Johnin Performance and IFN-γ Innovative Interpretative Criteria in the First and Second Groups

To evaluate the Se and Sp of the PPDJs using the different ICs, 128 animals were enrolled and followed up for 4 years. Among them, 67 from the first group were positive for at least one test for PTB, and 61 from the second group were always negative on the traditional tests for PTB. Table 2 shows the accuracy of the IFN-γ test according to the 12 ICs using PPDJA and PPDJB. Sp and Se values obtained with PPDJC are not shown because ATCC 19698 was used only for production control and method optimization.

**Table 2:** Specificity and sensitivity values obtained with 12 interpretative criteria of the IFN-γ test and cutoff values applied in the study for the diagnosis of Mycobacterium avium subsp. paratuberculosis (MAP) infected cattle.

**Interpretative criteria**		**SP (CI 95%)**	**SE (CI 95%)**	**Accuracy (A CI 95%)**	AUC^*^	**Y**
**1**	A-PBS > 0.05 = MAP	88.5% (80.3–96.7%)	85.1% (76.3–93.8%)	86.8% (80.7–92.7%)	0.868	73.6%
	Ja-PBS > 0.05 = MAP	93.4% (84.1–98.2%)	88.1% (77.8–94.7%)	90.6% (85.5–95.7%)	0.908	81.5%
	Jb-PBS > 0.05 = MAP	91.8% (81.9–97.3%)	100.0% (94.1–100.0%)	89.8% (84.5–95.1%)	0.899	79.9%
**2**	A-PBS > 0.1 = MAP	95.1% (89.5–100%)	76.1% (65.6–86.6%)	85.2% (78.9–91.4%)	0.856	71.2%
	Ja-PBS > 0.1 = MAP	100.0% (94.1–100.0%)	77.6% (65.8–86.9%)	88.3% (82.6–93.9%)	0.888	77.6%
	Jb-PBS > 0.1 = MAP	100.0% (94.1–100.0%)	80.6% (69.1–89.2%)	89.8% (84.5–95.1%)	0.903	80.6%
**3**	A-PBS > 0.2 = MAP	100.0% (94.1–100.0%)	58.2% (45.5–70.0%)	78.1% (70.8–85.3%)	0.791	58.2%
	Ja-PBS > 0.2 = MAP	100.0% (94.1–100.0%)	67.2% (54.6–78.2%)	82.8% (76.1–89.4%)	0.836	67.2%
	Jb-PBS > 0.2 = MAP	100.0% (94.1–100.0%)	62.7% (50.1–74.2%)	80.4% (73.5–87.4%)	0.813	62.7%
**4**	A-(2 ^*^ PBS) > 0 = MAP	90.2% (79.8–96.3%)	79.0% (67.4–88.1%)	84.3% (77.9–90.7%)	0.846	69.2%
	Ja-(2 ^*^ PBS) > 0 = MAP	98.4% (91.2–99.9%)	79.1% (67.4–88.1%)	88.2% (82.6–93.9%)	0.887	77.5%
	Jb-(2 ^*^ PBS) > 0 = MAP	93.4% (84.1–98.2%)	82.1% (70.8–90.4%)	87.5% (81.6–93.3%)	0.878	75.5%
**5**	A-(2 ^*^ PBS) > 0.04 = MAP	95.1% (86.3–99.0%)	73.1% (60.9–83.2%)	83.6% (77.1–90.1%)	0.841	68.2%
	Ja-(2 ^*^ PBS) > 0.04 = MAP	100.0% (94.1–100.0%)	71.6% (59.3–82.0%)	85.1% (78.9–91.3%)	0.858	71.6%
	Jb-(2 ^*^ PBS) > 0.04 = MAP	100.0% (94.1–100.0%)	70.2% (57.7–80.7%)	83.5% (77.0–90.0%)	0.85	70.2%
**6**	If PPDBov and/or PPDAv and/or PPDJ > 2 ^*^ PBS, then apply PPDBov/PPDAv and/or PPDBov/PPDJ					
	B/A ≤ 0.9 = MAP	90.2% (79.8–96.3%)	81.3% (69.5–89.9%)	83.6% (77.1–90.1%)	0.857	71.4%
	B/Ja ≤ 0.9 = MAP	100.0% (93.5–100.0%)	78.7% (66.3–88.1%)	82.8% (76.1–89.4%)	0.893	77.0%
	B/Jb ≤ 0.90 = MAP	94.9% (85.8–98.9%)	81.5% (70.0–90.1%)	85.1% (78.9–91.3%)	0.882	69.3%
**7**	If PPDAv and PPDJ > 2 ^*^ PBS, then apply PPDAv/PPDJ					
	A/Ja ≤ 0.90 = MAP	100.0% (93.5–100.0%)	68.4% (51.3–82.5%)	63.2% (54.8–71.7%)	0.842	68.4%
	A/Jb ≤ 0.90 = MAP	98.2% (90.3–99.9%)	73.7% (56.9–86.6%)	64.0% (55.6–72.4%)	0.859	73.2%
**8**	Ja/B > 1 = MAP	75.0% (62.7–85.5%)	88.0% (77.8–94.7%)	82.0% (75.2–88.7%)	0.817	63.0%
	Jb/B > 1 = MAP	69.0% (55.7–80.1%)	94.0% (85.4–98.4%)	82.0% (75.2–88.7%)	0.814	63.0%
**9**	Ja/A > 1 = MAP	90.2% (79.8–96.3%)	55.2% (42.6–67.4%)	71.8% (63.9–79.7%)	0.727	45.4%
	Jb/A > 1 = MAP	85.3% (73.8–93.0%)	53.7% (41.1–66.0%)	68.7% (60.6–76.8%)	0.695	39.0%
**10**	B/A > B/Ja = MAP	90.2% (79.8–96.3%)	55.0% (42.6–67.4%)	71.8% (63.9–79.7%)	0.727	45.0%
	B/A > B/Jb = MAP	85.0% (73.8–93.0%)	54.0% (41.1–66.0%)	68.7% (60.6–76.8%)	0.695	39.0%
**11**	Ja-A > 0.1 = MAP	100.0% (94.1–100.0%)	25.4% (15.5–37.5%)	60.9% (52.3–69.5%)	0.627	25.4%
	Jb-A > 0.1 = MAP	100.0% (94.1–100.0%)	28.9% (16.8–39.1%)	61.7% (53.1–70.2%)	0.634	28.9%
**12**	Ja-A > 0.05 = MAP	100.0% (94.1–100.0%)	35.0% (24.5–48.5%)	66.4% (58.1–74.6%)	0.679	35.0%
	Jb-A > 0.05 = MAP	98.4% (91.2–99.9%)	37.3% (25.8–50.0%)	66.4% (58.1–74.6%)	0.678	35.7%

PPD, purified protein derivative; PPDBov or B, bovine PPD; PPDAv or A, avian PPD; PPDJ, Johnin PPD; Ja, Johnin PPD from strain A; Jb, Johnin PPD from strain B; PBS, phosphate-buffered saline; SP CI 95%, specificity and confidence interval 95%; SE CI 95%, sensitivity and confidence interval 95%; A CI 95%, accuracy and confidence interval 95%; AUC, area under the ROC curve; ROC, receiver operating characteristic; Y, Youden's index. The asterisk symbol (^*^) is used to indicate the mathematical operation of multiplication.

Out of 71 animals that tested positive for traditional PTB tests, four were considered outliers (PBS > 0.150 OD) and were therefore excluded from the performance evaluation.

**Figure 1:**
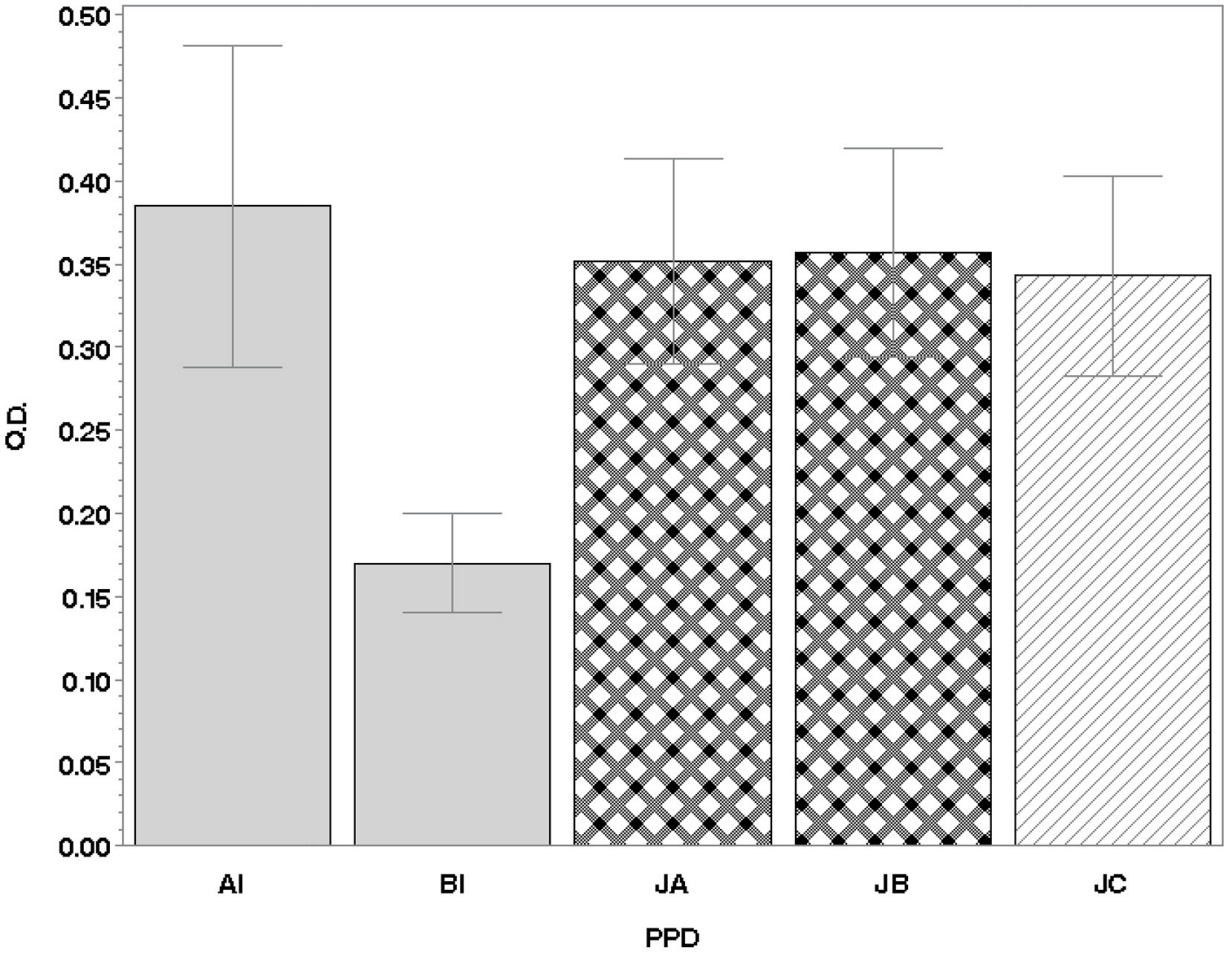
IFN-γ production in lymphocytes of 71 PTB-positive animals. Values are expressed as the mean of the differences between the OD_450nm_ of PPDs and the OD_450nm_ of PBS (±SD). IFN, interferon; PTB, paratuberculosis; PBS, phosphate-buffered saline; PPD, purified protein derivative; SD, standard deviation; OD, optical density; AI, Italian avian PPD; BI, Italian bovine PPD; JA, JB, JC, Johnins produced by the three strains of MAP: A and B (field strains) and C [strain American Type Culture Collection (ATCC) 19698].

The amount of IFN-γ produced by the PTB-positive subjects, in response to stimulation with the various PPDs, expressed in OD values, is represented graphically in Figure 1.

**Figure 2:**
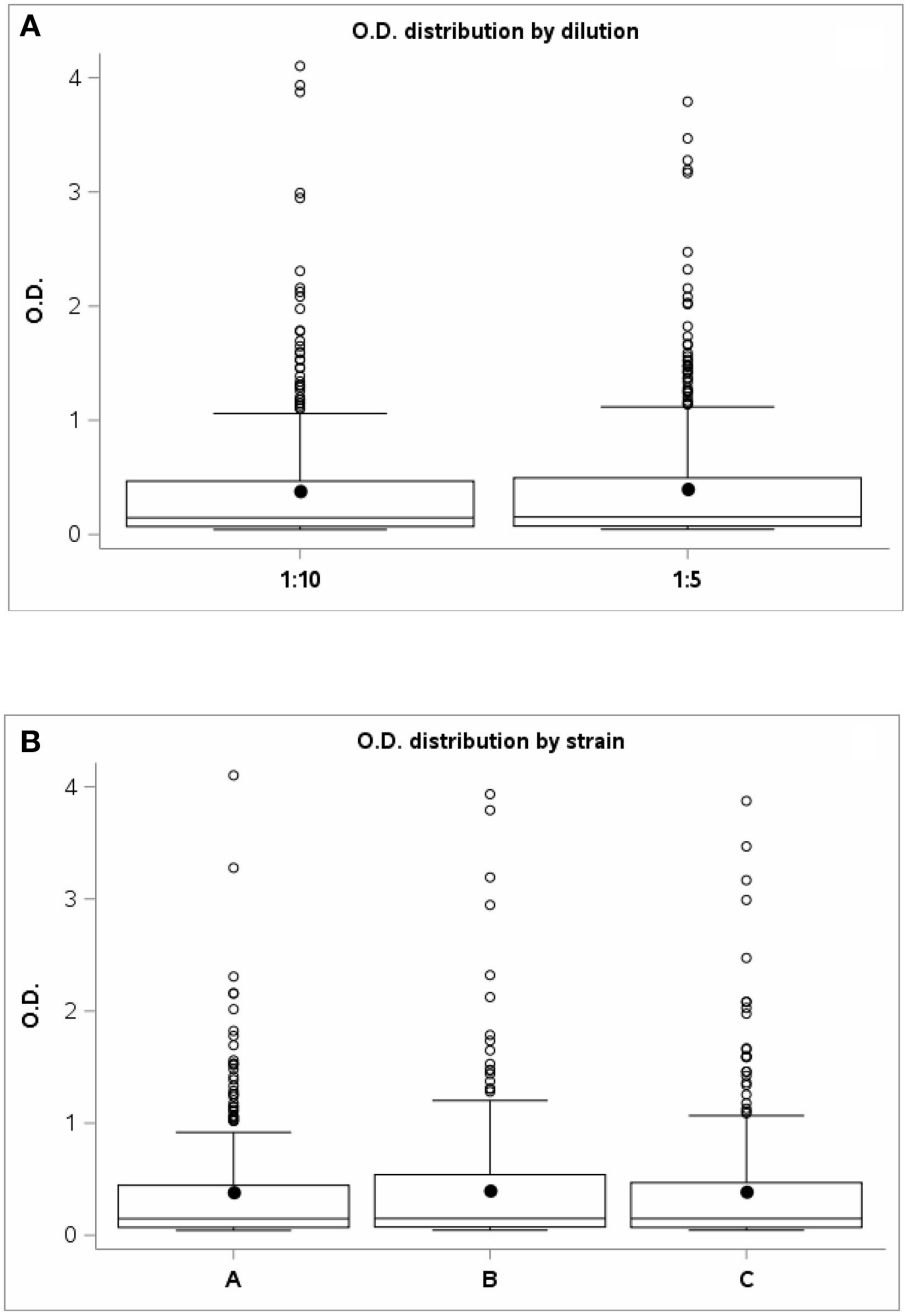
The graph shows the OD values distribution of PPD Johnins **(A)** for the two dilutions: 1:5 and 1:10 and **(B)** for strains A, B, and C. OD, optical density; PPD, purified protein derivative.

Regarding the assessment of PPDJ efficiency, analysis of variance showed no statistically significant differences between the mean OD 450 nm of the PPDJs and the dilution factor (F-test = 1.61; p = 0.2060) (Figure 2A) or for the strain factor (F-test = 0:37; p = 0.6907) (Figure 2B).

As shown in Table 2, among the 12 applied ICs, the PPDJs achieved the best performance within the first six ICs, with values of accuracy ranging from 90.6% (CI 95%: 85.5–95.7%) to 80.4% (CI 95%: 73.5–87.4%). The accuracy of the first six criteria was higher than that of the last six criteria, and the difference was statistically significant (binomial exact test, p <0.0001). The first criterion achieved a higher accuracy for criteria 3, 4, 5, and 6; and the difference was statistically significant for each criterion (binomial exact test, p <0.05). No statistically significant differences were observed between the accuracy of the first and second ICs; however, there were differences between the Se and Sp values. In particular, as shown in Table 2, PPDJB with the first criterion achieved better Se values (binomial exact test, p = 0.02; Se 100.0% CI: 94.1–100.0%), while PPDJB with the second criterion achieved the best Sp values (binomial exact test, p <0.0001; Sp 100.0% CI: 94.1–100.0%), but the accuracy was the same (89.8% CI: 84.5–95.1%).

Regarding the comparison of the diagnostic accuracy of PPDJs vs. PPDA, within the same criterion, statistically significant differences were observed in the second criterion (binomial exact test, p = 0.0397). In addition, in the second criterion, the Sp of PPDJs was higher than that of PPDA, and the difference was statistically significant (binomial exact test, p = 0.0381).

**Figure 3:**
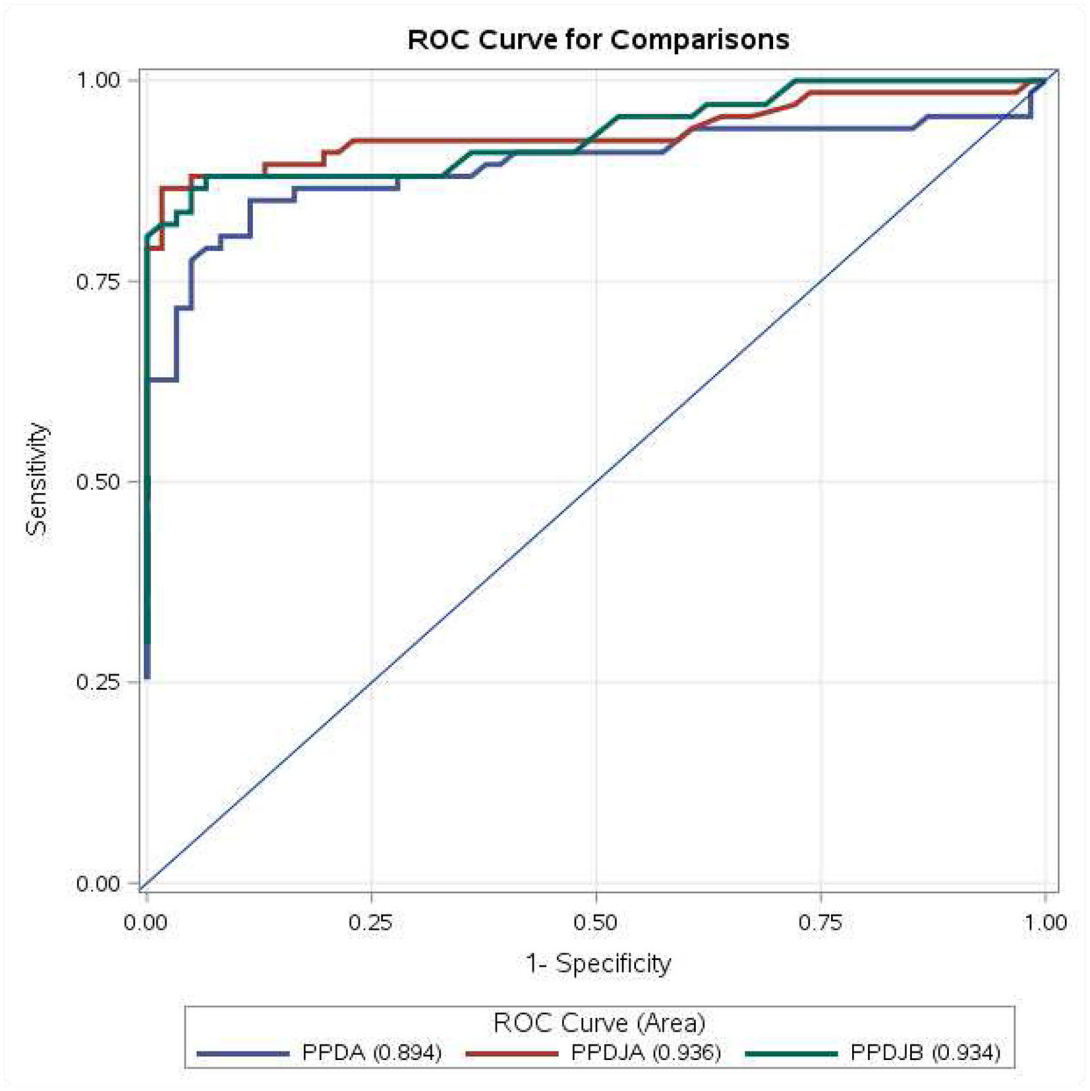
ROC curve for comparisons of PPDA-PBS, PPDJA-PBS, and PPDJB-PBS according to the first three interpretative criteria of the IFN-γ test applied in the study for the diagnosis of Mycobacterium avium subsp. paratuberculosis (MAP)-infected cattle. PPDJA and PPDJB showed higher AUC than PPDA, and the difference is statistically significant (*p* < 0.05). ROC, receiver operating characteristic; IFN, interferon; PPD, purified protein derivative; PPDA, avian PPD; PPDJA, PPD Johnin strain A; PPDJB, PPD Johnin strain B; AUC, area under the ROC curve.

Results of ROC analysis obtained with PPDA, PPDJA, and PPDJB according to the first three criteria are shown in Figure 3 and in Supplementary Tables 2, 3. Regarding the comparison between AUC of PPDJs and AUC of PPDA, the differences were statistically significant; in particular, the AUCs of PPDJA and of PPDJB were higher than those of PPDA, with a *p* < 0.001 and *p* < 0.05, respectively. No difference was observed between AUC of PPDJA and PPDJB, since DeLong's test for two correlated ROC curves was statistically not significant (*p* > 0.05).

As shown in Table 2, the IC that showed the best performance in terms of Se and Sp was derived from a comparison between the OD value obtained after PPD stimulation and the OD value of basal IFN-γ of each animal (PBS) (from the first to fifth criteria). All the ICs based on the simple difference or ratio between OD values relative to PPDs (from the eight to 12th criterion), not considering the baseline value of IFN-γ (OD value of PBS), elicited the worst results. The comparison between the criteria that provided difference and ratio showed that the criteria based on PPDs OD ratio, such as the sixth and seventh criteria, yielded numerous inconclusive outcomes (ND) for values in the cutoff range, despite good results in terms of Se and Sp. Furthermore, all the ICs that provided a difference or ratio between the PPDJs and the PPDA showed inefficacy, especially in terms of Se, with values ranging from 25.4% (CI 95%: 15.5–37.5%) to 55.2% (CI 95%: 42.6–67.4%).

### Evaluation of Purified Protein Derivatives Johnin Performance and IFN-γ Innovative Interpretative Criteria in the Third Group

In the third group, based on the data obtained from the application of the 12 criteria in the first and second groups on positive and negative PTB animals, respectively, the criteria with the best performance were used. In particular, in a herd with an ongoing bTB outbreak, the criteria that reported Sp values of 100% (second, third, and fifth ICs) were adopted with the aim of verifying the Sp of PPDJs.

Out of 87 animals that were negative to ELISA PTB tests, seven subjects were considered outliers (PBS > 0.150 OD) and therefore excluded from the performance evaluation. As shown in Table 3, for the second criterion (if PPDJ – PBS > 0.1 = MAP), the PPDJA at 1:10 dilution reached 95.0% Sp (CI 95%: 87.69–98.62%) and the PPDJB at 1:10 dilution provided 87.50% Sp (CI 95%: 78.21–93.84%). In the third criterion (if PPDJ – PBS > 0.2 = MAP), PPDJA at 1:10 dilution provided 100.0% Sp (CI 95%: 95.49–100.0%) and 95.0% Sp for PPDJB (CI 95%: 87.69–98.62%). Finally, in the fifth criterion (if PPDJ – 2 × PBS > 0.04 = MAP), the PPDJA at 1:10 dilution reached 96.25% Sp (CI 95%: 89.43–99.22%) and PPDJB reached 91.25% Sp (CI 95%: 82.80–96.41%). With the third criterion, PPDJA reached Sp values higher than those of the other two ICs (binomial exact test, *p* < 0.0001) and PPDA (binomial exact test, *p* = 0.0006).

**Table 3:** Specificity values obtained in the 3rd group of animals, adopting the 2nd, 3rd, and 5th interpretative criteria of the IFN-γ test.

**Interpretative criteria**		**N**	**TN**	**FP**	**%FP**	**SP**	**CI 95% SP**	
**2**	A-PBS > 0.1 = MAP	80	73	7	8.75%	91.25%	82.80%	96.41%
	Ja-PBS > 0.1 = MAP	80	76	4	5.00%	95.00%	87.69%	98.62%
	Jb-PBS > 0.1 = MAP	80	70	10	12.50%	87.50%	78.21%	93.84%
**3**	A-PBS > 0.2 = MAP	80	79	1	1.25%	98.75%	93.23%	99.97%
	Ja-PBS > 0.2 = MAP	80	80	0	0.00%	100.00%	95.49%	100.00%
	Jb-PBS > 0.2 = MAP	80	76	4	5.00%	95.00%	87.69%	98.62%
**5**	A-(2 ^*^ PBS) > 0.04 = MAP	80	74	6	7.50%	92.50%	84.39%	97.20%
	Ja-(2 ^*^ PBS) > 0.04 = MAP	80	77	3	3.75%	96.25%	89.43%	99.22%
	Jb-(2 ^*^ PBS) > 0.04 = MAP	80	73	7	8.75%	91.25%	82.80%	96.41%

*Specificity for IFN-y test criteria estimated on a sample of 80 animals from a Mycobacterium avium subsp. paratuberculosis (MAP) free herd with an ongoing bovine tuberculosis outbreak*.

*N, number of assessed animals; TN, true negative; FP, false positive; SP CI 95%, specificity and confidence interval 95%; A, avian PPD; Ja, Johnin PPD from strain A; Jb, Johnin PPD from strain B; PBS, phosphate-buffered saline*.

## Discussion

The IFN-γ test, in association with the tuberculin skin test, is used in many European countries for bTB diagnosis (63, 64). Both methods show the CMI response of the infected animals following stimulation with mycobacterial antigens, PPDB and PPDA (58, 63, 82). The immunologic evaluation to identify animals infected with MB is often limited by cross-reactions observed in animals exposed to other species of mycobacteria, particularly those belonging to the *Mycobacterium avium* complex (MAC), mainly MAP (86, 92).

In a single intradermal comparative cervical tuberculin (SICCT) test and in the IFN-γ release assay, a reaction to PPDA can identify animals affected by PTB (58, 82, 93–95). For this reason, in cattle, the IFN-γ test has also been used recently for the early diagnosis of PTB, although the Sp of the method varies from 67 to 94%, depending on the type and amount of PPDs used, and particularly on the IC of the test (57, 60).

With the aim of producing new batches of PPDJ obtained from the field strains of MAPs common in our territory and to develop a more sensitive and specific IFN-γ test for the early diagnosis of MAP infection, three experimental PPDJs were produced at IZSUM, and different IFN-γ ICs were evaluated.

From the first contact with mycobacteria, the immune response is characterized by a complex series of events aimed at controlling the infection before it can compromise homeostasis in the organism (45). In this context, the secretion of pro-inflammatory cytokines, such as IFN-γ, is involved in the containment of infection caused by mycobacteria (48) and can be detected only by the IFN-γ test. However, the humoral immune response, detectable by ELISA, appears only in the late stage when the disease is clinically manifested (60). The animals become infected at a young age, but the clinical PTB form does not occur until 2–3 years, probably because of the control role of innate and CMI, genetic susceptibility of the animal, and environmental factors (45, 47, 48, 50, 75). Therefore, it is strategically advantageous to decide in which categories include animals with a positive reaction to the IFN-γ test and tested negative to traditional tests for MAP infection diagnosis (49, 75). The production of IFN-γ by lymphocytes, after stimulation with PPDs, indicates an “immunological memory” and therefore a previous contact with a mycobacterium, and in the case of the PTB, the presence of a MAP infection. Thus, as an oversimplification, an IFN-γ-positive reactor is a MAP-infected animal that is probably in the stage of infection in which the animal, through CMI, keeps the pathogen under control and avoids its spread, as reported by recent studies (44). At this stage of infection, the animal, even if infected, does not yet eliminate MAP with feces and does not present any antibodies. To date, it is not predictable how long the animal will remain in this stage of infection. Certainly, in a herd with a high PTB prevalence, an IFN-γ-positive reactor could be a possible future MAP shedder and will therefore need to be checked more often than other cattle. However, if this IFN-γ-positive reactor will never test positive in conventional tests, it could be a subject able to contain the MAP infection, which will never develop the disease. It will be an important challenge for future studies to understand whether these animals with these characteristics will be identified as resistant or resilient PTB cattle (49, 75).

As stated earlier, the “infected” animals that do not yet shed MAP and do not yet show clinical signs are the most difficult category to identify with ELISA, fecal culture, and qPCR, due to low antibody production and low or absent MAP shedding. In addition, the “subclinical infected” animals may contaminate the environment, but their detection by serological test and the direct MAP identification has lower probability of success, representing a significant challenge for the control and management of PTB (57). “Affected” animals often present a clinical form and shed large quantities of MAP, representing the main source of infection, and are frequently positive in serological tests and can be easily detected by MAP isolation and biomolecular approaches.

In the present study, through a 4-year follow-up, it was possible to define the different stages of MAP infection in cattle of the first group from three PTB-affected herds, using new Italian Johnins in the IFN-γ assay. The obtained data represent the first assessment of the performance of experimental Italian PPDJs in the IFN-γ test and the first evidence of their ability to detect MAP-infected animals by adopting different ICs.

The critical aspect of this study was the comparison between two different diagnostic approaches, one based on the IFN-γ test and the other based on traditional tests (ELISA, fecal culture, and qPCR). The first approach is able to detect animals in the preliminary stages of MAP infection, while the second is useful when affected subjects are in the advanced stages of the disease or already have the clinical form of PTB. Therefore, the Se and Sp of the IFN-γ assay are related to the tests adopted to define the “positive animal.” These parameters are calculated on the basis of tests applied at the different stages of infection, in animal producing antibodies against MAP and/or in animal shedding MAP, while the IFN-γ test reveals infected animals that generally do not yet produce antibodies and do not yet shed MAP in their feces. This may have a negative effect on the performance of the IFN-γ test.

Simultaneously to the performance evaluation of the new PPDJs, different criteria for the PTB IFN-γ test interpretation were developed and compared, particularly IC based on the difference between OD values and IC based on the OD ratio (Table 1). Moreover, different cutoff values have been applied to each criterion, some of which are often used for the diagnosis of bTB in cattle and buffalo (72, 92, 96).

The PPDJs achieved the best performance within the first six ICs, among the 12 applied ICs (Table 2), with values of accuracy ranging from 90.6% (CI 95%: 85.5–95.7%) to 80.4% (CI 95%: 73.5–87.4%). In particular, the accuracy achieved by the PPDJs adopting the first six criteria resulted in a higher accuracy than that using the last six criteria, and the difference was statistically significant (binomial exact test, *p* < 0.0001). In addition, both PPDJs with the first criterion achieved a higher accuracy related to values reached adopting IC from the third to sixth, and the difference was statistically significant for each criterion (binomial exact test, *p* < 0.05).

From the perspective of the field use of PPDJs, we wanted to identify the IC that would maximize their performance, both in terms of Se, as in the case of the first criterion (PPDJB Se 100.0%, CI: 94.1–100.0%), and in terms of Sp, as in the case of the second IC (PPDJs Sp 100.0%, CI: 94.1–100.0%). In fact, even though no statistically significant differences were observed between the first and second ICs, in terms of accuracy, PPDJB with the first criterion achieved better Se values (binomial exact test, *p* = 0.02), while PPDJB with the second criterion achieved the best Sp values (binomial exact test, *p* < 0.0001), despite the accuracy being equal. As stated before, the Sp and Se are greatly affected by the tests used to detect the positive animal. On the basis of the six best ICs, in the first group, among 16 subjects with negative outcomes in the three PTB conventional tests, six were IFN-γ positive. The follow-up allowed monitoring of PTB progression, and five animals became PTB positive to traditional tests. In particular, two animals that reacted to both PPDA and PPDJs had PTB-positive bacterial culture 3 months later and also to the ELISA 10 months later. Among the three bovines reactive only to PPDJs, two animals became ELISA-positive 6 months later and a one bovine 18 months later. In the first step, these animals were considered “false positive” by statistical analysis, but the IFN-γ test with the six best ICs detected and unveiled MAP-infected cattle earlier than did the other traditional tests (Supplementary Table 1 and Supplementary Figure 1).

In terms of the mean IFN-γ production following stimulation with PPDJs and PPDA, there were no statistically significant differences (Figure 1) except in the second criterion, where the Sp of PPDJs was higher than the Sp of PPDA, and the difference was statistically significant (binomial exact test *p* = 0.0381). However, the analysis of the ROC curves of the PPDJA, PPDJB, and PPDA, according to the first three criteria (Figure 3 and Supplementary Tables 2, 3), shows that there were no statistical difference between AUC of PPDJA and of PPDJB, since DeLong's test for two correlated ROC curves was statistically not significant (*p* > 0.05). Instead, it is important to highlight that AUC of PPDA was different from PPDJA and PPDJB and that the difference was statistically significant (*p* < 0.05).

This result can be explained since MAP is part of the MAC; therefore, the remarkable similarities between PPD extracted from MAP and PPD extracted from MA do not permit the use of one against the other. However, in the criteria where both PPDJs and PPDA were used (Table 2), the first ones reached better values in terms of accuracy and proved to be more specific, particularly when used with the second criterion. Moreover, the comparison between the two PPDJs did not reveal statistically significant differences, even if the PPDJB seemed to be more sensitive and the PPDJA seemed more specific (Table 2, first and second ICs). These aspects, related to the performance of PPDJs and the robustness of IC validation, could be improved by enrolling more animals in future investigations. In addition, a further evolution of our study could be the use of recombinant antigens, peptides, or proteins from MAP, to increase the Sp of the IFN-γ test (97). These antigens have already been widely used in the IFN-γ test for the diagnosis of bTB in bovines (98, 99) and buffalo (92), and the most widely used antigens are ESAT6/CFP10, or other antigen cocktails, with the aim of increasing the Sp of the IFN-γ test. In the literature, the results obtained with recombinant proteins of MAP have not been completely satisfactory (100). Nevertheless, we are evaluating possible candidates for future inclusion in the lymphocyte stimulation phase of the IFN-γ test, such as the more promising MAP2698C (62) and MAP0586C (101) proteins. However, it should be noted that our experimental PPDJs achieved Sp values of 100%, without compromising Se values and therefore maintaining high accuracy values, when the second, third, and fifth ICs were adopted (Table 2) and when used with the third criterion in a bTB outbreak (Table 3). However, recombinant antigens tend to favor Sp but penalize Se because of their high discriminating power.

Although valid criteria were highlighted in this study, in particular the first and the second, in our opinion, the main aspect is the adoption of different criteria in relation to the PTB status in each farm. In particular, it is advisable to use those criteria with a major Se, such as the first criterion, in herds with a high prevalence of PTB; on the contrary, apply more specific IC, such as the second criterion, in herds with low PTB prevalence, similar to the protocol described by Keck et al. (73) used in France during the bTB eradication plans for cattle from 2003 to 2014.

In this regard, it was of great interest to include in the experiment a particularly problematic herd, the third enrolled group, which consisted of animals from a herd with a bTB outbreak at the time of the survey. In addition, animals have always been brought to pasture in the summer months; hence, they are subject to infections due to atypical mycobacteria, particularly those belonging to the MAC. Therefore, in these animals, non-specific reactions in the PTB IFN-γ test related to possible cross-reactions to MB and MAC were predictable. In these cases, it was useful to assess the Sp of PPDJs to avoid false-positive outcomes for MAP in animals that have always been negative on ELISA tests for PTB. In particular, in this third group, with the aim of verifying the Sp of PPDJs, the second, third, and fifth ICs were adopted because they reached Sp values of 100% (CI 95%: 94.1–100.0%) in the other groups. PPDJA achieved Sp values of 100% (CI 95%: 95.49–100%) when adopted in the third criterion, and the difference with other ICs was statistically significant (binomial exact test, *p* < 0.0001). Always regarding the third criterion, PPDJA has shown higher Sp than PPDA, and the difference was statistically significant (Binomial exact test, *p* = 0.0006). Other authors (100) included a group of animals from a bTB outbreak in their assessment of the efficiency of the IFN-γ test for PTB diagnosis and did not find the same performance achieved in the present study. The authors concluded that the IC that they used led to several inconclusive results, and the use of a PPDJ would have resulted in a more precise classification of the animals within the bTB outbreak. Therefore, the use of the PPDJs investigated in the present study and the IC with the best performance could be recommended in herds where bTB and PTB co-infection is suspected, a scenario that could be an additional challenge for us.

In conclusion, the use of PPDJs and the interpretation of the IFN-γ test with the first or second criterion achieved high performance in the identification of MAP-infected cattle. Therefore, it can be assumed that the IFN-γ test could be a useful tool for identifying early subclinical MAP-infected animals, managing cattle infected or exposed to MAP, and monitoring younger calves within a herd.

## Conclusions

The IFN-γ test should be used for the early diagnosis of MAP infection, and it can be efficiently used to detect pre-maturely MAP-infected subjects within a herd. Considering that infected cattle may never shed MAP or show clinical signs of PTB, this would allow veterinarians and farmers to decide together about the future of infected animals. The future of the animal, that is, to keep or to cull, must be considered carefully based on PTB prevalence on a farm. In particular, in herds with high PTB prevalence, culling IFN-γ positive reactors could mean eliminating animals that are “controlling” the infection, and paradoxically these animals may be “resistant” to the disease, and as such, should be kept in the herd.

On the contrary, in herds with low PTB prevalence or that are PTB-free, an animal that reacts positively to the IFN-γ test is definitely an animal that has been exposed to MAP or has contracted the infection; therefore, it has to be removed in order to maintain the low PTB or PTB-free status in the herd. Furthermore, the IFN-γ test can be considered as an additional test for animals that may be admitted to the herd, to avoid the introduction of MAP-infected subjects, especially in herds that have already eradicated the disease. Hence, the IFN-γ test will provide an additional diagnostic tool that farmers could adopt voluntarily to reach and preserve health status certification regarding PTB.

## Data Availability Statement

The raw data supporting the conclusions of this article will be made available by the authors, without undue reservation.

## Author Contributions

PM: conceptualization. SC, ADi, CS, LC, PP, SCo, NV, ADo, and PM: methodology. NV: software. SC, ADi, and PM: validation. NV, LP, CS, MCi, LC, and PM: formal analysis. SC, ADi, CS, MCi, LC, MB, PP, SCo, and PM: investigation. SC, ADi, MB, ADo, and PM: resources. SC, ADi, NV, and PM: data curation. SC, ADi, MT, LP, and PM: writing original draft preparation. SC, ADi, MT, LP, MCa, and PM: writing review and editing. SC, NV, ADi, LP, and PM: visualization. SC, ADi, NV, MT, LP, MCa, ADo, and PM: supervision. All authors contributed to the article and approved the submitted version.

## Conflict of Interest

The authors declare that the research was conducted in the absence of any commercial or financial relationships that could be construed as a potential conflict of interest.
