# Supplementary material for: Early Detection of Mycobacterium avium subsp. paratuberculosis Infected Cattle: Use of Experimental Johnins and Innovative Interferon-Gamma Test Interpretative Criteria
Source: Front Vet Sci. 2021 May 14;8:638890. doi: 10.3389/fvets.2021.638890 (PMC8160102; doi:10.3389/fvets.2021.638890)
Supplement: Supplementary file 1 [file Data_Sheet_1.PDF]

Supplementary Material

Table S1 Results of paratuberculosis (PTB) diagnosis with traditional test considered in parallel (serology/polymerase chain reaction/culture) and with interferon (IFN)-γ test derived from the six best interpretative criteria.

		PTB		
		**P**	**N**	**Total**
**IFN-γ**	**P**	59	6	**65**
	**N**	12	10	**22**
	**Total**	**71***	**16**	**87**

*P, positive; N, negative. *In the statistical analysis, in order to estimate the sensitivity, out of 71 PTB-positve subjects, 4 animals were excluded due to high optical density values of the phospate buffered solution.*

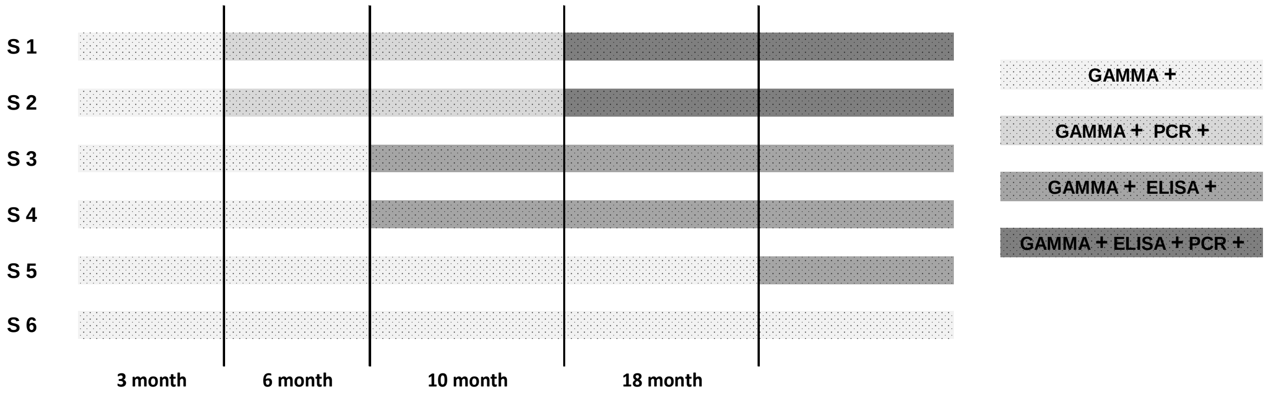

Figure S1. Timeline in six subjects positive only on interferon (IFN)-γ test, five of which became positive, during the follow-up, to serology (gamma + / ELISA +); to polymerase chain reaction (gamma + / PCR +); to serology and polymerase chain reaction (gamma + / ELISA + / PCR +) in the next 18 months.

Table S2 ROC Association Statistics according to DeLong's test, relative to data reported in the Figure 3.

ROC Model	Mann-Whitney				Somers' D	Gamma	Tau-a
	AUC	Standard Error	95% Wald Confidence Limits				
**PPDJA**	0.9358	0.0246	0.8876	0.9839	0.8715	0.8735	0.4382
**PPDJB**	0.9341	0.0228	0.8894	0.9787	0.8681	0.8722	0.4365
**PPDA**	0.8942	0.0317	0.8321	0.9563	0.7884	0.7901	0.3964

PPDJA, PPD Johnin strain A; PPDJB, PPD Johnin strain B; PPDA, Avian PPD; ROC, receiver operating characteristic; AUC, area under the curve

Table S3 ROC Contrast Estimation and Testing Results by Row according to DeLong's test, relative to data reported in the Figure 3.

Contrast	Estimate	Standard Error	95% Wald Confidence Limits		Chi-Square	Pr > ChiSq
**PPDJA - PPDA**	0.0416	0.0127	0.0166	0.0666	10.6580	0.0011
**PPDJB - PPDA**	0.0399	0.0174	0.00578	0.0740	5.2535	0.0219

PPDJA, PPD Johnin strain A; PPDJB, PPD Johnin strain B; PPDA, Avian PPD; ROC, receiver operating characteristic.
